# PhyloFusion—Fast and Easy Fusion of Rooted Phylogenetic Trees into Rooted Phylogenetic Networks

**DOI:** 10.1093/sysbio/syaf049

**Published:** 2025-07-17

**Authors:** Louxin Zhang, Banu Cetinkaya, Daniel H Huson

**Affiliations:** Department of Mathematics and Centre for Data Science and Machine Learning, National University of Singapore, #07-25, Block S17, 10 Lower Kent Ridge Road, Singapore 119076, Singapore; Institute for Bioinformatics and Medical Informatics, and Excellence Cluster “Controlling Microbes to Fight Infection”, University of Tübingen, Sand 14, Tübingen 72076, Germany; Institute for Bioinformatics and Medical Informatics, and Excellence Cluster “Controlling Microbes to Fight Infection”, University of Tübingen, Sand 14, Tübingen 72076, Germany

**Keywords:** Algorithms, phylogenetic networks, phylogenetics, reticulate evolution, software

## Abstract

Unrooted phylogenetic networks are commonly used to represent evolutionary data in the presence of incompatibilities. Although rooted phylogenetic networks offer a more explicit framework for depicting evolutionary histories involving reticulate events, they are reported less frequently, probably due to a lack of tools that are as easily applicable as those for unrooted networks. Here, we introduce PhyloFusion, a fast and user-friendly method for constructing rooted phylogenetic networks from sets of rooted phylogenetic trees. The resulting networks have the tree-child property. The algorithm accommodates trees with unresolved nodes—often resulting from the contraction of low-support edges—as well as some degree of missing taxa. We demonstrate its application to the analysis of functionally related gene groups and show that it can efficiently handle data sets comprising tens of trees or hundreds of taxa. An open source implementation of PhyloFusion is available as part of the SplitsTree app: https://www.github.com/husonlab/splitstree6. All data available here: https://doi.org/10.5061/dryad.k3j9kd5h5

Rooted phylogenetic trees are used to explicitly represent the evolutionary history of a set of species or other taxa, in terms of speciation events, at branching nodes (i.e., nodes with two or more children), and often involving some measure of time or evolutionary distance, as branch lengths (Felsenstein 2004). However, when reticulate events, such as speciation-by-hybridization, horizontal gene transfer, or reassortment, have played a significant evolutionary role, then these require the use of a rooted phylogenetic network in which reticulate nodes (i.e., nodes with two or more parents) explicitly represent putative reticulate events (Koblmüller et al. 2007; Pimentel et al. 2013; Ottenburghs et al. 2017; Rosser et al. 2024).

One approach to obtaining such networks is to first compute rooted phylogenetic trees for two or more genes and then to compute a rooted phylogenetic network *N* that contains or “displays” all the input gene trees (see Fig. 1), and minimizes the number of reticulations contained in the network, or, more precisely, minimizes the hybridization number, which is defined as


\begin{eqnarray*}
h(N)=\sum _{v \ne \rho }\big (\mbox{in-degree}(v)-1\big ),
\end{eqnarray*}

where the sum is over all nodes *v* except the root node $\rho$. (A full account of definitions, algorithmic procedures, and formal results is provided in the Supplementary Appendix.) Note that only *reticulate* nodes of in-degree ≥2 contribute to the value, whereas *tree nodes* of in-degree 1 do not contribute.

**Figure 1.**
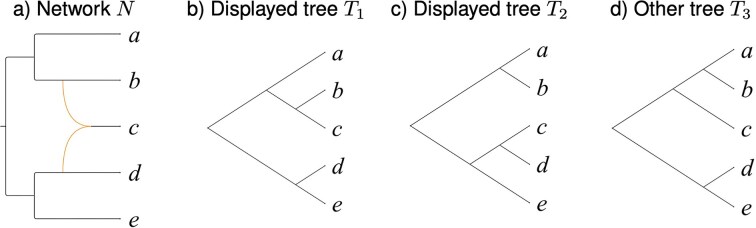
Tree display. (a) A rooted phylogenetic network *N* with hybridization number $h(N) = 1$, containing a single reticulation node with two incoming reticulation edges. Using the upper reticulation edge, the network displays tree $T_1$ shown in (b); using the lower edge, it displays tree $T_2$ shown in (c). The network does not display tree $T_3$ shown in (d).

In mathematical phylogenetics, such a network has been referred to as a “hybridization network” (Bordewich and Semple 2007) due to one possible interpretation of the reticulate nodes as corresponding to hybridization events. However, in practice, gene-tree discordance can also be due to other reasons, such as errors in tree inference, rooting and resolution, or incomplete lineage sorting, say. The computational problem of determining the minimum hybridization number for two trees is known to be NP-hard (Bordewich and Semple 2005).

Several algorithms with associated software have been developed to address this problem (Wu 2010; Piovesan and Kelk 2013; van Iersel et al. 2014, 2022; Mirzaei and Wu 2016; Borst et al. 2022). Four recent ones are given below:

- Autumn (Huson and Linz 2018) takes as input two rooted phylogenetic trees that may include multifurcations (i.e., nodes with out-degree 3 or more) and differing taxon sets (i.e., allowing missing taxa), and it outputs all possible rooted networks that display the trees with the minimum hybridization number. As an exact algorithm, it is limited to small instances. It is implemented in Dendroscope (Huson and Scornavacca 2012) and SplitsTree (Huson and Bryant 2024).
- TreeKnit (Barrat-Charlaix et al. 2022), developed for analyzing viral reassortment, also takes two rooted phylogenetic trees (allowing multifurcations) and heuristically computes a rooted phylogenetic network minimizing the hybridization number. It follows a greedy strategy, identifying compatible regions in both trees. It is available as a Julia package.
- ALTS (Zhang et al. 2023) processes multiple rooted phylogenetic trees, but requires them to be bifurcating with no missing taxa. It computes a tree-child rooted phylogenetic network (as defined in Supplementary Section A.2), heuristically minimizing the hybridization number. The method encodes trees as sequences, aligns them, and decodes the aligned sequences into a network. It is available as a standalone C program.
- FHyNCH (Bernardini et al. 2024) takes multiple rooted phylogenetic trees (allowing multifurcations and missing taxa) and computes a rooted phylogenetic network with “orchard” properties (as defined in Supplementary Section A.3), heuristically minimizing the hybridization number. It employs cherry picking and a machine-learning-based heuristic. It is available as a Python package.

There are several other approaches to computing networks from biological data (e.g., Stolzer et al. 2012; Solís-Lemus and Ané 2016; Wen et al. 2018; Allman et al. 2019, 2025; Rabier et al. 2021; Kong et al. 2024). Approaches that aim to infer a phylogenetic network directly from sequence data or gene trees, using principles such as maximum parsimony, maximum likelihood, or Bayesian inference, are appealing because they extend well-established frameworks for tree inference to the more general case of networks. However, they are typically limited to very small data sets, involve many model parameters that can be difficult to estimate reliably, and their application can be computationally challenging.

In this paper, we introduce PhyloFusion, an algorithm that extends ALTS by supporting trees with multifurcations and missing taxa. PhyloFusion is designed to facilitate the exploration of reticulate evolutionary scenarios using a limited set of informative gene trees, typically involving up to 100 taxa. It is particularly suited for cases where the number of required reticulate events remains small enough to allow interpretability.

Our simulations suggest that PhyloFusion produces networks with lower hybridization numbers than TreeKnit and FHyNCH for such data sets. By contrast, FHyNCH performs better on larger, more complex data sets with many trees, taxa, missing taxa, and contracted edges (Bernardini et al. 2024). This is an “out-of-the-box” comparison, with all algorithms run using default settings, and any of the three can likely be optimized to outperform the others.

One practical advantage of PhyloFusion is that it is explicitly designed for use by biologists and is provided within the interactive SplitsTree app (Huson and Bryant 2024), whereas tools such as ALTS, TreeKnit, and FHyNCH are provided as standalone command-line tools with nontrivial installation and setup requirements, available as source code on GitHub.

The networks computed by PhyloFusion satisfy the tree-child property, meaning that every nonleaf node has at least one child that is not the product of reticulation (Cardona et al. 2009). This constraint reflects the expectation that each ancestral lineage gives rise to some extant lineage through ordinary divergence.

To illustrate the intended use of PhyloFusion, Figure 2 presents several rooted phylogenetic networks computed from 43 photosynthesis-related chloroplast gene trees of 17 water lilies, using multiple sequence alignments from Gruenstaeudl (2019). In each case, network computation takes only a few seconds. Although we do not propose that reticulate evolution plays a major role in this data set, it serves as a practical example of how PhyloFusion can be used to explore rooted phylogenetic networks as a means of representing the evolutionary history of related gene sets in an intuitive and efficient manner.

**Figure 2.**
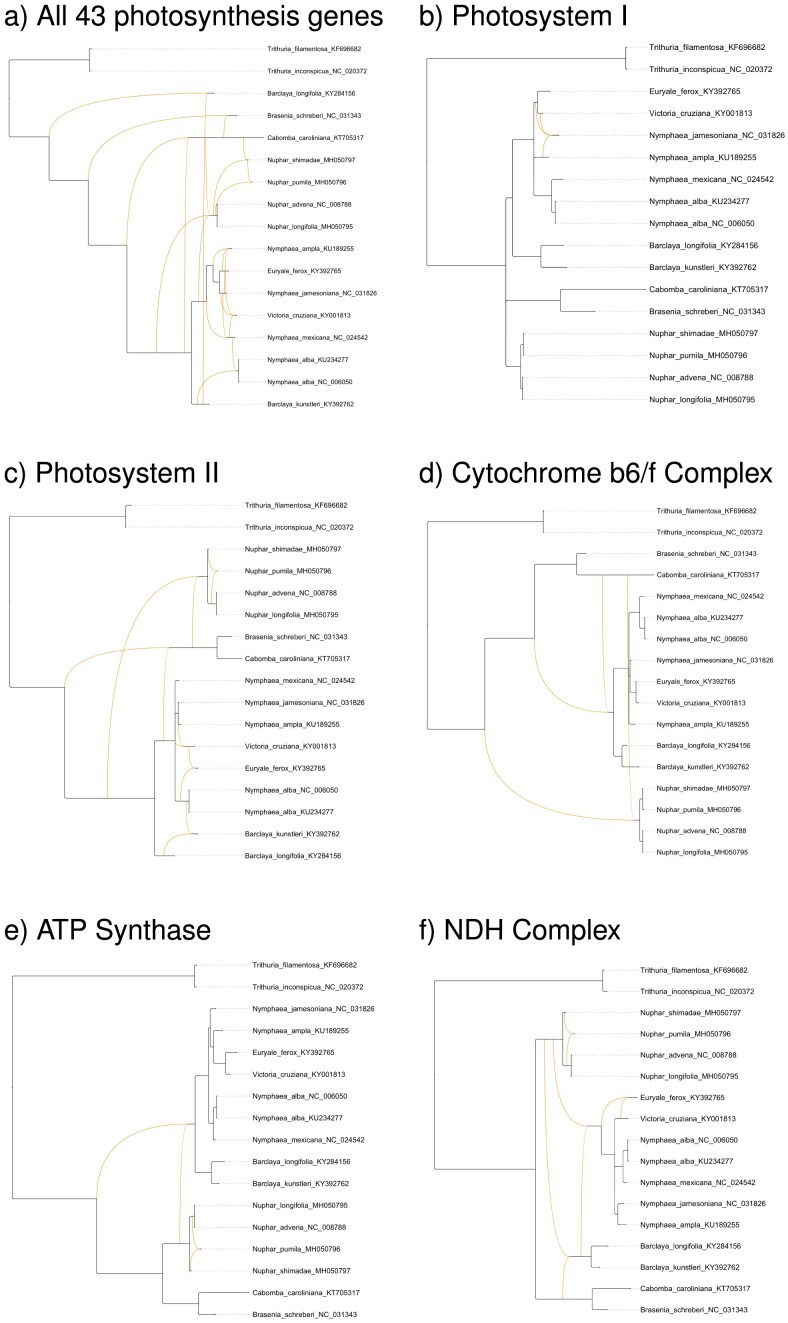
Rooted networks. For rooted chloroplast gene trees from 17 water lily species (Gruenstaeudl 2019), we present the following rooted phylogenetic networks, computed using the PhyloFusion algorithm with a minimum bootstrap threshold of 70%. (a) A network for all 43 photosynthesis-related gene trees ($h=13$). (b) A network for all five *psa* (Photosystem I) gene trees ($h=2$). (c) A network for all 15 *psb* (Photosystem II) gene trees ($h=6$). (d) A network for all six *pet* (Cytochrome b6/f Complex) gene trees ($h=2$). (e) A network for all six *atp* (ATP Synthase) gene trees ($h=2$). (f) A network for all 11 *ndh* (NDH Complex) gene trees ($h=4$). Here, $h=h(N)$ denotes the hybridization number of the displayed network *N*.

## Material and Methods

Let $T_1,\dots ,T_k$ be an input set of rooted, bifurcating phylogenetic trees on a set of taxa $X=\lbrace x_1,\dots ,x_n\rbrace$. In the following, we will always assume that the root node has out-degree one. This can always be arranged by placing a new root above the original root, if the original root has a higher out-degree.

The ALTS (“Align LTS”) algorithm proceeds in a number of steps.

First, choose a fixed total ordering $\pi$ on the set of taxa, that is, $x_{\pi (1)} < x_{\pi (2)} < \dots < x_{\pi (n)}$.

**Figure 3.**
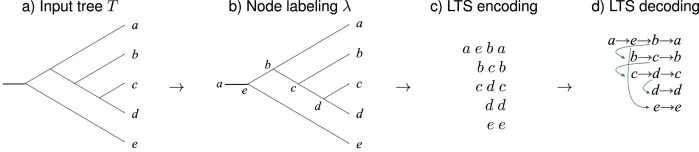
LTS encoding and decoding of a tree. Here, $X=\lbrace a,\dots ,e\rbrace$ and we use the taxon ordering *a < b < c < d < e*. (a) Input is a rooted phylogenetic tree *T* on *X*. (b) Each node *u* is given a label $\lambda (u)$. The root is labeled by the smallest taxon, in this case *a*. The label $\lambda (\ell )$ of each leaf $\ell$ is the taxon that it represents. Every internal node *u* with children *v* and *w* is labeled by $\lambda (u)=\max \big (\min \lbrace x\mbox{~on leaf below~}v\rbrace ,\min \lbrace x\mbox{~on leaf below~}w\rbrace \big )$, the larger of the smallest leaf labels below *v* and below *w*, respectively. (c) Each taxon appears exactly twice, once as an internal label and once as a leaf label. For each taxon *x* in ascending order, we extract the LTS sequence of all labels on the path from its internal occurrence to its leaf occurrence. (d) To decode the LTS encoding shown in (c), represent each label by a node and connect each node to its immediate successor in the same row. In addition, connect any internal node with label *y* to the first node in the row corresponding to the LTS of *y*. To obtain the final tree, remove any “through nodes” that have in-degree one and out-degree one.

Second, based on $\pi$, for each input tree $T_i$, compute a labeling $\lambda$ of all nodes in the tree, as described in Figure 3b. This labeling gives rise to the *LTS encoding* of $T_i$ that consists of one *LTS* sequence $LTS_i(x)$ of labels per taxon *x*, listed in the given order of the taxa (Fig. 3c) (see Supplementary Algorithm C.4). Any rooted tree can be recovered from its LTS encoding using the decoding procedure explained in Figure 3d (see Supplementary Algorithm C.5).

**Figure 4.**
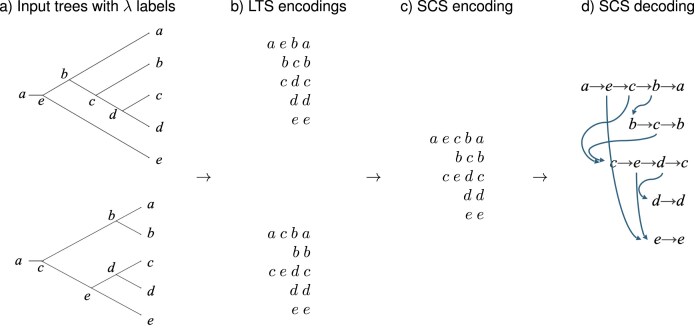
ALTS algorithm. Here, the taxon set is $X=\lbrace a,\dots ,e\rbrace$ and the ordering is *a* < *b < ··· < e*. (a) Compute the node labeling $\lambda$ on each input tree. (b) Extract the LTS encoding for each tree. (c) For each taxon *x*, compute a shortest common supersequence of the corresponding LTS sequences to obtain the SCS encoding. (d) Decoding gives rise to a rooted phylogenetic network that displays all input trees.

Third, for each taxon *x*, determine a shortest common supersequence $SCS(x)$ of all its LTS sequences *LTS*_1_(*x*), …, *LTS_k_*(*x*) (one per input tree) and list all such supersequences in the given order of the taxa, to obtain a new list of sequences that we call the *SCS encoding* of the input trees (see Fig. 4c). The SCS encoding can be decoded as discussed above and the result will be a rooted phylogenetic network that displays all input trees (see Fig. 4d; Zhang et al. 2023).

In this construction, a reticulation node can only arise at the beginning of an LTS sequence. Hence, every node is connected to a leaf by a path consisting of tree nodes (given by the LTS sequence that contains the node). This implies that the resulting network has the *tree-child property*.

The ALTS algorithm is based on a number of mathematical insights (Zhang et al. 2023).

Lemma 1. Let *T* be a rooted, bifurcating phylogenetic tree on *X* and $\pi$ a fixed ordering on *X*, and assume, as always in this paper, that the root has out-degree 1. When calculating the $\lambda$ labeling and the LTS encoding for *T*, the following statements hold:

1. Each taxon appears exactly twice in the $\lambda$ labeling, once on an internal node (including the root) and once on a leaf.
2. For each taxon *x*, except the smallest one, there exists exactly one taxon *y* for which *x* appears in the LTS for *y*. Moreover, *y* comes before *x* in the taxon ordering $\pi$.
3. The decode procedure recovers the original input tree.
4. If additional occurrences of taxa are inserted into the LTS encoding of *T* in such a way that statement 2 remains true, then decoding will result in a rooted phylogenetic network *N*, rather than a tree, and that network displays *T*.

Zhang et al. (2023) prove the following.

Theorem 2. The *ALTS algorithm* produces a tree-child network that displays all input trees and minimizes the hybridization number, if one considers all possible $n!$ orderings on *X* and if one solves a shortest common supersequence optimally.

The *ALTS heuristic* considers only a subset of all possible taxon orderings and uses a heuristic to compute a shortest common supersequence for more than two sequences, an NP-hard problem (Maier 1978).

## The PhyloFusion Algorithm

A key advantage of the ALTS heuristic is its conceptual simplicity, as its central computation is multiple sequence alignment. However, the requirement that input trees must be bifurcating and include all taxa limits its practical applicability. Here, we introduce PhyloFusion, an extension of the ALTS algorithm, which accepts input trees with multifurcations and missing taxa and computes a rooted phylogenetic network that displays the input trees while heuristically aiming to minimize the hybridization number.

A rooted phylogenetic tree *N* is said to (soft) *display* a rooted phylogenetic tree *T*, if *T* can be obtained from *N* by deleting nodes and edges, and/or contracting edges (for more details, see Supplementary Section B). We say that *N hard displays* a tree *T*, if each multifurcation in *T* also appears in *N*.

Given a collection of rooted phylogenetic trees that may include multifurcations, the PhyloFusion algorithm computes a rooted phylogenetic network *N* that hard displays all input trees and minimizes the hybridization number $h(N)$ (as defined in Supplementary Section C). As we will see, the PhyloFusion algorithm follows modifications of the same steps as the ALTS algorithm. Similar to ALTS, a heuristic is derived by considering a subset of taxon orderings and using a profile alignment heuristic to compute shortest common super-hypersequences.

Unfortunately, hard display is not of interest in phylogenetics because a multifurcation represents a lack of resolution rather than a multiway speciation event. A network that hard displays a set of trees may require a higher hybridization number than a network that soft displays the trees. Note that missing taxa can also give rise to reticulations that appear in a network that hard displays the input trees but are not necessary in a network that soft displays them.

In light of this, although the core PhyloFusion algorithm is designed to compute a minimum hybridization network that hard displays a given set of input trees, practical applications in evolutionary biology usually require targeting the more flexible concept of soft display. Accordingly, we first present the PhyloFusion algorithm in the context of hard display, and then introduce additional heuristics that, when used in conjunction with PhyloFusion, are tailored to address the soft display setting.

Let $T_1,\dots ,T_k$ be an input set of rooted, phylogenetic trees on a set of taxa $X=\lbrace x_1,\dots ,x_n\rbrace$. We do not assume that the trees are bifurcating and we allow missing taxa. The latter implies that any input tree $T_i$ may be a *partial tree* (Huson et al. 2012), that is, a phylogenetic tree on its own subset of taxa $X_i \subseteq X$, for $i=1,\dots ,k$. We sometimes refer to this as a phylogenetic tree on *X* with missing taxa.

The PhyloFusion algorithm follows the same steps as the ALTS algorithm, but differs in some of the details. First, choose a fixed total ordering $\pi$ on the set of taxa, that is, $x_{\pi (1)} < x_{\pi (2)} < \dots < x_{\pi (n)}$.

**Figure 5.**
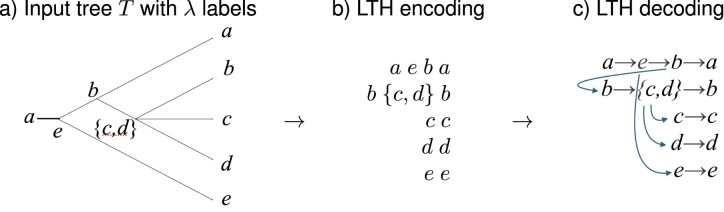
LTH encoding and decoding of a multifurcating tree. Here, the taxon set is $X=\lbrace a,\dots ,e\rbrace$ and the ordering is *a < b < c < d < e*. (a) The root, leaves, and bifurcating nodes are labeled as shown in Figure 3b. Let *u* be a multifurcating node with children $v_1,\dots ,v_t$, and for each such child $v_i$, let $m(v_i)$ denote the smallest taxon below $v_i$, respectively. We set $\lambda (u)=\lbrace m(v_1),\dots , m(v_k)\rbrace \setminus \min \lbrace m(v_i)\rbrace$, the set of the smallest taxa below each of the children of *u*, except the smallest one. (b) LTHs for the taxa *a, b, c, d*, and *e*, shown from top to bottom. (c) Decoding is performed as described in Figure 3d, with the additional rule that if the label of an internal node contains multiple taxa, then the node is connected to the first node in each row that corresponds to an LTH associated with one of the taxa in the label.

**Figure 6.**
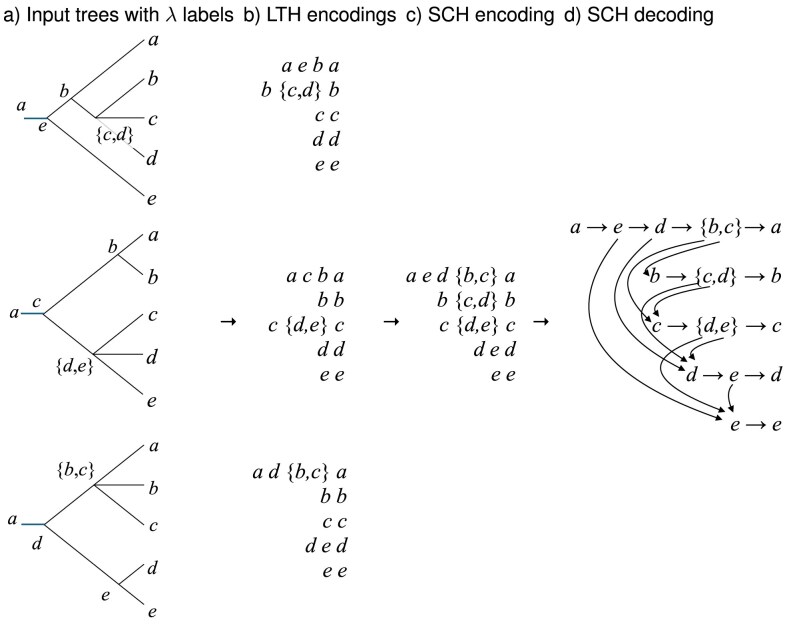
PhyloFusion algorithm. (a) For a fixed ordering on *X*, here *a < b < ··· < e*, compute the node labeling $\lambda$ on each input tree. (b) Extract the LTH encoding for each tree. (c) For each taxon *x*, compute a shortest common hyper-supersequence of the corresponding LTH sequences to obtain the SCH encoding. (d) Decoding gives rise to a rooted phylogenetic network that hard displays all input trees.

Second, based on $\pi$, for each input tree $T_i$, compute a labeling $\lambda$ of all nodes in the tree, as indicated in Figure 6a, using Supplementary Algorithm C.6. This labeling gives rise to the *LTH encoding* of $T_i$ that consists of one *lineage taxon hypersequence*  $LTH_i(x)$ of labels per taxon *x*, listed in the given order of the taxa (Fig. 6b). (Any rooted, possibly multifurcating, phylogenetic tree can be recovered from its LTH encoding using the decoding procedure explained in Fig. 5c and Supplementary Algorithm C.8.)

Third, for each taxon *x*, determine a shortest common super-hypersequence $SCH(x)$ of all its LTH sequences *LTH*_1_(*x*), …, *LTH_k_*(*x*) (one per input tree) and list all such super-hypersequences in the given order of the taxa, to obtain a new list of sequences that we call the *SCH encoding* of the input trees (see Fig. 6c). Note that the LTH sequences are “hypersequences” whose elements might be sets rather than single taxon labels, and in this case two elements may be matched with each other if one contains the other. The SCH encoding can be decoded as discussed above and the result will be a rooted phylogenetic network that contains all input trees (Fig. 6d), using Supplementary Algorithm C.9.

We have the following result.

Theorem 3. Given an input collection of rooted phylogenetic trees $T_1,\dots , T_k$ on *X*, allowing multifurcations and missing taxa. The *PhyloFusion algorithm* produces a tree-child network *N* that hard displays all input trees and minimizes the hybridization number, if one considers all possible $n!$ orderings on *X* and if one solves all shortest common supersequence problems optimally.

Theorem 3 is restated as Supplementary Theorem D.12 and proven in Supplementary Section D.2. The rationale is as follows. First note that, as in the case of the ALTS algorithm, the resulting network has the tree-child property (Supplementary Proposition C.10), because a reticulation can only appear at the beginning of an LTH sequence.

Let *T* be a tree on *X* with LTH encoding *L*. Note that, by construction, creating an augmented encoding $L^{\prime }$ by inserting additional taxon labels into *L*—that is, a super-hypersequence of *L*—will only give rise to additional nodes and edges in the corresponding decoding, and for any edge $(p,q)$ in the decoding of *L* there will exist an edge $(p,q)$ or a directed path from *p* to *q* in the decoding of $L^{\prime }$. This implies that the network computed by the PhyloFusion algorithm will hard display all input trees (see Supplementary Theorem D.12).

To see that an optimal network can be obtained, let *N* be a tree-child network that displays the set of input trees. Note that we can construct an ordering on the taxa using the structure of the network such that the LTH $S_i$ of each taxon $x_i$ in the network is the common super-hypersequence of the corresponding LTHs in the trees. So, if *N* has the minimum hybridization number $h(N)$, then $S_i$ must be a shortest common *super-hypersequence* of the corresponding LTHs in the trees (see Supplementary Proof of Theorem D.12).

The *PhyloFusion heuristic* is based on two modifications. It does not consider all $n!$ possible orderings on the taxa. Instead, the heuristic first considers all *n* orderings in which one of the taxa is placed at position 1 of the ordering and then fixes a taxon at position 1 that minimizes the hybridization number obtained (using the original ordering of the remaining taxa). Next, in a similar fashion, the taxon at position 2 of the ordering is determined. This is repeated until all taxa have been placed into the ordering. In this way, we consider only $O(n^2)$ orderings in total. This procedure is rerun a fixed number of rounds (by default 10), or until no further improvement of the hybridization number was obtained in a fixed number of rounds (by default 3).

Second, for any two sequences, PhyloFusion computes a shortest common super-hypersequence efficiently using the Needleman–Wunsch algorithm (Needleman and Wunsch 1970) aimed at minimizing the alignment score, using a match score of 0, a mismatch score of $\infty$, and a gap score of 1. To compute a shortest common super-hypersequence for more than two sequences, PhyloFusion uses the progressive alignment heuristic (Feng and Doolittle 1987).

**Figure 7.**
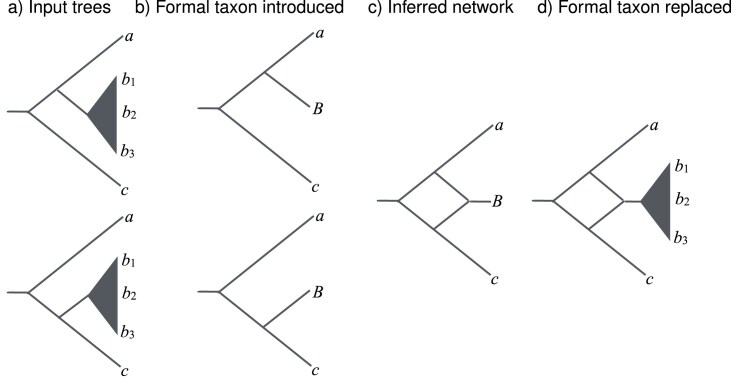
Recursion step. (a) Two input trees sharing a common cluster or subtree on *b*_1_, *b*_2_, and *b*_3_. (b) The common cluster is replaced by a formal taxon *B*. (c) The PhyloFusion algorithm is applied to the trees on taxa *a, B*, and *c*. (d) The formal node *B* is replaced by the original tree (in the case of a subtree reduction) or computed network (in the case of a cluster reduction) on *b*_1_, *b*_2_, and *b*_3_.

Similar to the implementation of the ALTS heuristic and the autumn algorithm, our implementation of the PhyloFusion heuristic is recursive. In each recursive step, the input is a collection of input trees and the output is a network that displays the trees. At the beginning of the recursion, we attempt to perform a subtree reduction (representing a set of congruent subtrees by a formal taxon) and, optionally a cluster reduction (splitting the problem into two subproblems, one above the cluster, in which the cluster is represented by a formal taxon, and the other in the cluster). See Figure 7 and Supplementary Section D.3 for a more detailed discussion.

## Additional Heuristics

As discussed above, the core PhyloFusion algorithm aims at determining a rooted phylogenetic network *N* that *hard displays* an input set of rooted phylogenetic trees and minimizes the hybridization number $h(N)$. The presence of multifurcations, or missing taxa, can both lead to a hybridization number larger than required by a network that *soft displays* the input trees. As mentioned, soft display is the more relevant concept in evolutionary applications, and so some additional work is required.

There are two preprocessing steps that address this that can be undertaken at the beginning of each recursive call in the algorithm. First, mutually refine all input trees with respect to each other. This will remove some of the multifurcations in the input. Second, remove any tree that is a subtree of another input tree. This will reduce some instances of missing taxa.

We now describe two additional heuristics that operate on the LTH sequences and may apply when the two preprocessing steps do not.

### Refinement Heuristic

**Figure 8.**
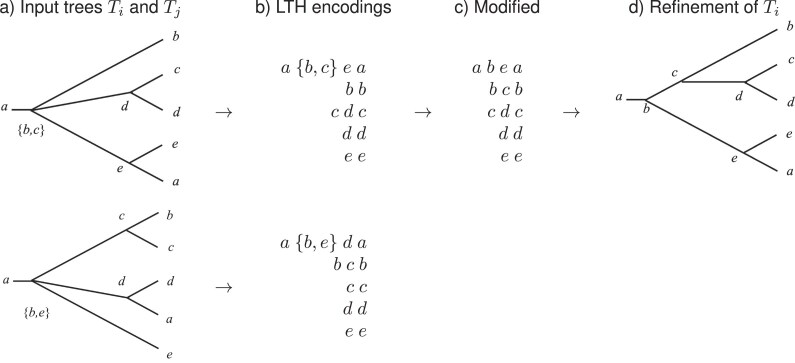
Refinement heuristic. (a) Two input trees $T_i$ (top) and $T_j$ (bottom), labeled using the ordering *a* < *b* < *···* < *e*. (b) With the notation in the text, choose $x=a$ with $LTH_i(x)=(a,\lbrace b,c\rbrace ,e,a)$, $E=\lbrace b,c\rbrace$, $R=\lbrace c\rbrace$ and $y=b$. We have $LTH_i(y)=(b,b)$ and $LTH_j(y)=(b,c,b)$. (c) For $T_i$, we set $LTH_i(x)=(a,b,e,a)$ and $LTH_i(y)=(b,c,b)$, which corresponds to refining tree $T_i$ as shown in (d).

Assume that *x* is a taxon whose LTH sequence $LTH_i(x)$, for some tree $T_i$, contains an element *E* of size $\ge 2$ (see Fig. 8). Choose $y\in E$ and $R\subseteq E\setminus \lbrace y\rbrace$. If none of the elements of *R* appears anywhere else in the LTH of *x* in any input tree, then we search for a second tree $T_j$ such that *y* appears in $LTH_j(x)$ and $LTH_j(y)$ contains all the elements of *R*. If found, we remove the elements of *R* from *E* and insert the set *R* at the beginning of $LTH_i(y)$, the LTH sequence of *y* in tree $T_i$. This operation corresponds to refining the node corresponding to *E* along the edge toward the taxon *y*. See Supplementary Appendix for further details.

Note that the example shown in Figure 8 cannot be addressed using mutual refinement of the two trees in a preprocessing step.

### Add-Leaf Heuristic

**Figure 9.**
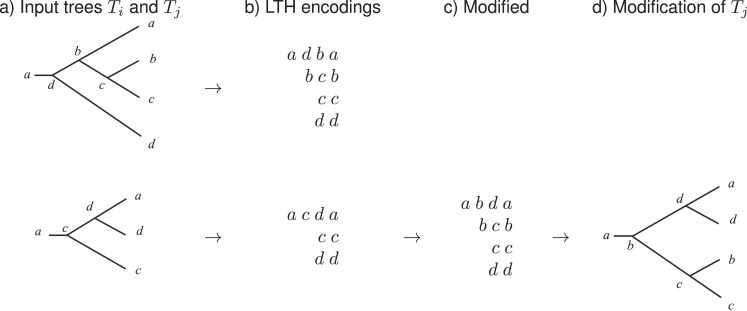
Add-leaf heuristic. (a) The two input trees $T_i$ (top) and $T_j$ (bottom), labeled using ordering *a* < *b* < *c* < *d*. (b) The corresponding LTH encodings. The tree $T_j$ is missing taxon *b*. (c) With the notation in the text, set $x=b$, $y=c$, and $w=a$. Then modify the encoding for $T_i$, as described in the text, to obtain the encoding shown in (c), which corresponds to the modified tree shown in (d).

Assume that taxon *x* is contained in input tree $T_i$ and not in input tree $T_j$, for $i\ne j$ (see Fig. 9). If *x* is contained in the LTH sequence $LTH_i(w)$ for some other taxon $w<x$ in $T_i$, and if there exists a taxon $y>x$ that is contained in the LTH sequence $LTH_j(w)$ for *w* in $T_j$, and that is also contained in the LTH sequence $LTH_i(x)$ for taxon *x* in $T_i$, then replace *y* by *x* in $LTH_j(w)$, and set the LTH sequence for *x* and $T_j$ to $LTH_j(x)=(x,y,x)$. This has the effect of adding the taxon *x* to the input tree $T_j$. See Supplementary Section D.5 for further details.

Note that in the example shown in Figure 9, the smaller tree $T_j$ is not a subtree of the larger tree $T_i$, and so, in this case, the preprocessing step in which we remove any input tree that is a subtree of some other input tree does not apply.

## Results

To ensure ease of use, we have implemented PhyloFusion in the SplitsTree app (Huson and Bryant 2024). PhyloFusion is a heuristic for determining a rooted phylogenetic network *N* that (soft) displays a given set of rooted input trees, allowing both multifurcations and missing taxa, and that seeks to minimize the hybridization number $h(N)$.

Two recently published heuristics address the same problem, TreeKnit (Barrat-Charlaix et al. 2022) and FHyNCH (Bernardini et al. 2024). Although FHyNCH allows the same input as PhyloFusion, the TreeKnit program only accepts two input trees, and it does not accept missing taxa. All three approaches are heuristics and can be tuned to either produce networks with lower hybridization scores, or to run faster, depending on the application. We compare the three methods as provided, using default settings. FHynCH requires training to run in machine-learning mode, and we used the trained model that was used in Bernardini et al. (2024).

**Figure 10.**
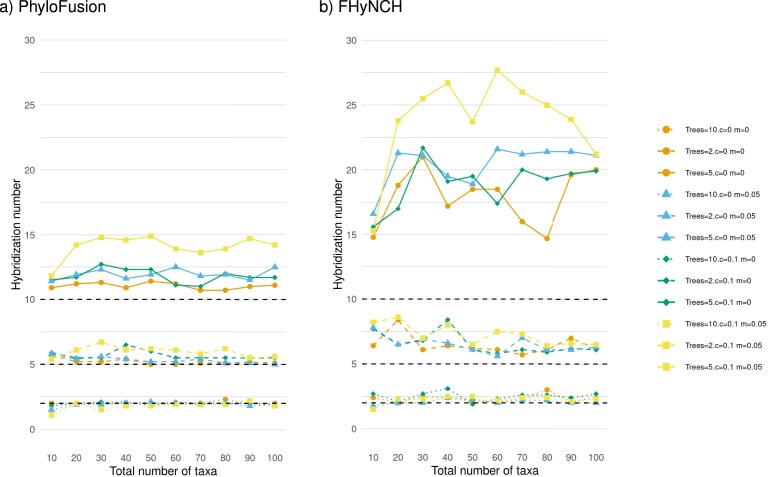
For input sets of 2, 5, and 10 trees (each tree obtained from a common seed tree by applying one rSPR), we plot the hybridization number of the networks computed by (a) PhyloFusion and (b) FHyNCH as a function of the number of taxa in the trees. Each point represents the average over 10 samples. In the legend, *c* and *m* denote the proportion of contracted internal edges and missing taxa in each input tree, respectively. Dashed lines indicate the total number of rSPRs applied to the input. Both programs were run with default settings. PhyloFusion required up to 2 min for the largest samples, whereas FHyNCH completed each run in under 10 s.

In Figure 10, we plot the hybridization number obtained using both PhyloFusion and FHyNCH, for different numbers of taxa, input trees, and proportions of contracted edges and missing taxa. To produce a sample set of input trees for each datapoint in this figure, we began with a large *background tree* containing 500 taxa. Each sample was generated in a two-step process: First, we selected a random subset of taxa and extracted a corresponding *seed tree*, defined as the rooted subtree induced by those taxa within the background tree. Second, we generated each input tree by applying a single rooted subtree prune and regraft (rSPR) operation to the seed tree, with both the prune and regraft locations chosen at random. We use the rSPR operation because of its close relationship to hybridization networks (Baroni et al. 2005). We then contracted a proportion *c* of randomly selected internal edges and/or removed a proportion *m* of randomly selected taxa from each input tree. As a result, each input tree differs from the seed tree by at most a single rSPR operation, along with possible contractions or taxon removals. Consequently, any phylogenetic network that soft displays all *k* input trees will have hybridization number at most *k*. Each point in the plot represents the hybridization number averaged over 10 different samples. Both methods were provided with exactly the same sets of input trees. The plots suggest that PhyloFusion produces networks whose hybridization numbers are much lower than those obtained using FHyNCH for these data.

**Figure 11.**
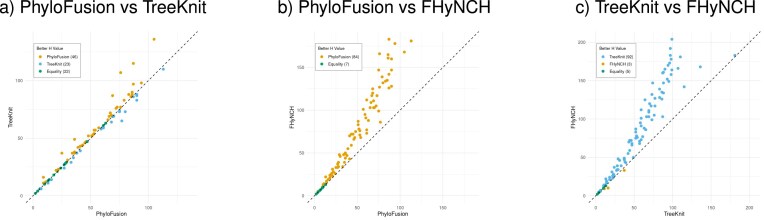
For 100 pairs of input trees, ranging from 10 to 400 taxa with an rSPR distance equal to 10% of the number of taxa, we plot (a) the hybridization number obtained by TreeKnit as a function of that obtained by PhyloFusion, (b) the hybridization number obtained by FHyNCH as a function of that obtained by PhyloFusion, and (c) the hybridization number obtained by FHyNCH as a function of that obtained by TreeKnit.

In Figure 11, we compare PhyloFusion, TreeKnit, and FHyNCH, on 100 pairs of input trees, ranging from 10 to 400 taxa, no contracted internal edges in either tree, and an rSPR distance equal to 10% of the number of taxa. In terms of speed, both TreeKnit and FHyNCH required only seconds for a single data set, even when the two trees contain hundreds of taxa. PhyloFusion took several minutes to process some of the larger data sets and timed out (after 15 min) on nine trees containing 400 taxa. In terms of the hybridization number, PhyloFusion performs better than TreeKnit in many cases, and both PhyloFusion and TreeKnit perform much better than FHyNCH in nearly all cases.

In Bernardini et al. (2024), FHyNCH was trained and evaluated on a broad range of data sets, spanning 10–1000 taxa, 10–100 trees, hybridization numbers from 10 to 100, and up to 20% missing taxa and/or contracted edges. Although these parameter ranges exceed those intended for PhyloFusion, we tested our software on a subset of these data, using 54 trees per parameter combination. The results, summarized in Table 1, suggest that FHyNCH is better suited for handling highly complex data sets (when appropriately trained).

**Table 1.** For a subset of the simulated data from Bernardini et al. (2024), we compare the performance of PhyloFusion and FHyNCH across different combinations of missing taxa percentages (rows) and contracted edge percentages (columns)

	0% contracted edges		10% contracted edges		20% contracted edges	
Missing taxa	PhyloFusion	FHyNCH	PhyloFusion	FHyNCH	PhyloFusion	FHyNCH
0%	**43**	8	25	**26**	23	**29**
10%	25	**27**	18	**35**	19	**34**
20%	17	**36**	9	**45**	13	**40**

Note: The table reports the number of cases (out of 54) in which each method achieved a lower hybridization number, with the better percentage highlighted in bold.

We also ran FHyNCH on the real data sets presented in Figure 2. These data sets consist of rooted trees inferred using IQ-Tree (Minh et al. 2020) from aligned chloroplast gene sequences of 17 water lily species (Gruenstaeudl 2019), with all edges below 70% bootstrap support contracted. In all six cases, PhyloFusion produced a lower hybridization number than FHyNCH, with differences ranging from 1 to 3.

## Discussion

To summarize, the basic PhyloFusion algorithm operates in four main steps:

- select a total ordering of all taxa;
- extract the LTH sequence for each taxon in each input tree;
- compute a shortest common super-hypersequence for each taxon; and
- decode the set of hypersequences to construct a network that hard displays all input trees.

When the input trees contain multifurcations or missing taxa, additional heuristics are applied to further reduce the number of reticulate nodes while maintaining the property that the network soft displays the input trees. The algorithm is applied recursively, first attempting a subtree reduction—or optionally, a cluster reduction—before executing other steps.

Reticulate events are crucial in evolution, yet rooted phylogenetic networks are rarely reported in biological literature. A key reason is the lack of widely adopted tools for their computation.

There has been much work on developing methods to infer rooted phylogenetic networks using principles such as maximum parsimony, maximum likelihood, or Bayesian inference. However, these approaches are considerably more computationally demanding than their tree-based counterparts, due to the vastly larger space of possible topologies and the need to estimate additional parameters, such as inheritance probabilities at reticulation nodes.

A promising approach, as explored here and in other recent studies, is to infer rooted networks from carefully computed phylogenetic trees. Several algorithms have been developed for this task. Among them, the ALTS algorithm is particularly attractive due to its conceptual simplicity.

PhyloFusion extends ALTS to accommodate trees with contracted edges and missing taxa. However, this extension aims at “hard display” rather than “soft display,” which does not align with the needs of biologists. To mitigate this, we introduced several heuristics, though our experimental evaluation suggests that handling large numbers of unresolved nodes and missing taxa remains challenging for our implementation.

With PhyloFusion, we aim to provide biologists with a practical tool for exploring the utility of rooted phylogenetic networks in evolutionary scenarios involving a moderate number of reticulate events, such as speciation-by-hybridization. To facilitate its adoption, we have integrated PhyloFusion into the SplitsTree app (Huson and Bryant 2024), ensuring ease of installation and use.

## Supplementary Material

syaf049_Supplemental_Files

## Data Availability

An open source implementation of PhyloFusion is available as part of the SplitsTree app: https://www.github.com/husonlab/splitstree6. The data used in this paper are available here: https://doi.org/10.5061/dryad.k3j9kd5h5
