# Supplementary material for: PhyloFusion—Fast and Easy Fusion of Rooted Phylogenetic Trees into Rooted Phylogenetic Networks
Source: Syst Biol. 2025 Jul 17;75(1):88–99. doi: 10.1093/sysbio/syaf049 (PMC12805670; doi:10.1093/sysbio/syaf049)
Supplement: syaf049_Supplemental_Files [file syaf049_supplemental_files.zip › appendix.pdf]

PhyloFusion- Fast and easy fusion of rooted phylogenetic trees into rooted phylogenetic networks

Louxin Zhang
Department of Mathematics and Centre for Data Science and Machine Learning,
National University of Singapore, Singapore 119076, Singapore,

Banu Cetinkaya and Daniel H. Huson*
Institute for Bioinformatics and Medical Informatics, and
Excellence Cluster "Controlling Microbes to Fight Infection",
University of Tübingen, 72076 Tübingen, Germany

July 5, 2025

Appendix

A Basic concepts and notation

A.1 Rooted phylogenetic trees and networks

Let X be a finite set of taxa. A *rooted phylogenetic tree* on X is a directed tree with a distinguished node called the root, satisfying the following conditions:

- All edges are directed away from the root.
- The root has in-degree 0 and out-degree 1.
- Each leaf (a node with out-degree 0) has in-degree 1 and is uniquely labeled by an element of X.
- Every internal node (i.e., nodes that are neither the root nor leaves) has in-degree 1 and out-degree at least 2.

A tree node is said to be a *multifurcation* or *non-resolved* if its out-degree is at least 3. A rooted phylogenetic tree is said to be *binary* if it does not contain any multifurcations.

A *rooted phylogenetic network* on X is a directed acyclic graph with a distinguished node called the root, satisfying the following conditions [Huson et al., 2012]:

*Corresponding author: daniel.huson@uni-tuebingen.de

- All edges are directed away from the root.
- The root has in-degree 0 and out-degree 1.
- Each leaf (a node with out-degree 0) has in-degree 1 and is uniquely labeled by an element of X.
- Every internal node (i.e., nodes that are neither the root nor leaves) has either in-degree 1 and out-degree at least 2, or in-degree at least 2 and out-degree 1.

In a rooted phylogenetic network on X, internal nodes with in-degree 1 and out-degree at least 2 are called *tree nodes*, while internal nodes with in-degree at least 2 and out-degree 1 are called *reticulation nodes*. Each reticulation node represents a reticulation event.

The *hybridization number* of a rooted phylogenetic network N is defined as:

$$h(N) = \sum_{v \neq \rho} (d^-(v) - 1), \tag{1}$$

where the sum is over all nodes that are not the root ρ, and $d^-(v)$ is the in-degree of v.

A rooted phylogenetic network is said to be *binary* if all trees nodes have out-degree 2 and all reticulation nodes have in-degree 2.

Consider a rooted phylogenetic network N. Let u and v be two nodes in N. We say that u is a *parent* of v, and v is a child of u, if there exists an edge from u to v. We say that u is an *ancestor* of v if there is a directed path from the root to v that passes through u. We also say that v is *below* u if u is an ancestor of v.

For the remainder of this text, we will identify each leaf with its label, and simply say that x is a leaf for each $x \in X$. In addition, we will simply refer to a rooted (binary) phylogenetic tree as a (binary) tree, and to a rooted (binary) phylogenetic network as a (binary) network.

A.2 Tree-child networks

We say that N is *tree-child* if every non-leaf node in N has at least one child that is a tree node [Cardona et al., 2009].

Let N be a tree-child network on X with k reticulation nodes. Removing all incoming arcs to each of the k reticulation nodes in N disconnects the network into $k+1$ connected components, each of which is a subtree rooted at either the root of N or at a reticulation node. These components are called the *tree components* of N. An important property of these tree components is that every leaf of each tree component is a network leaf.

Let r_0 denote the root of N and let the k reticulation nodes be $r_1, r_2, \cdots, r_k$. Since N is acyclic and rooted, we can topologically sort its nodes so that for any two reticulation nodes r_i and r_j, r_i appears before r_j in the ordering if a parent of r_j is in the tree component rooted at r_i. This ordering on $r_0, r_1, \cdots r_k$ induces an ordering on the tree components.

A.3 Cherry-picking and orchard networks

Let N be a network on X. An ordered pair of leaves (x, y) is considered a *cherry* if x and y share a common parent p. It is considered a *reticulation cherry* if the parent $p(x)$ of x is a reticulation node and $p(x)$ and y share a common parent.

Reducing a cherry (x, y) in N consists of the following two operations:

- Delete leaf x from N;
- Merge $p(y)$ with y if x and y are the only children of $p(y)$ and label the resulting node with y.

Reducing a reticulation cherry (x, y) in N consists of the following three operations:

- Remove the edge between $p(x)$ and $p(y)$.
- Delete x and label $p(x)$ with x if $p(x)$ has become an in-degree 1 node.
- Delete y and label $p(y)$ with y if $p(y)$ has become a out-degree 1 node after the first operation.

Reducing a cherry or a reticulation cherry (x, y) results in a network. We denote this network by $N[(x, y)]$. A sequence S of ordered pairs of leaves

$$(x_1, y_1), (x_2, y_2), \cdots, (x_k, y_k)$$

is called a *cherry-picking sequence* if (x_j, y_j) is a cherry or reticulation cherry in $N[(x_1, y_1)][(x_2, y_2)] \cdots [(x_{i-1}, y_{i-1})]$ for each $j \leq k$.

A network is *orchard* if there is a cherry-picking sequence S such that the network obtained by repeatedly reducing the pairs of S in order from N is a tree with one leaf [Erdős et al., 2019].

B Hard-display and soft-display of trees in a network

B.1 Hard-display of trees

The *subdivision* of an edge (u, v) in a network replaces that edge with two edges (u, w) and (w, v) by inserting a new node w between u and v. The reverse operation of *suppression* removes a node w that has exactly one parent u and exactly one child v, replacing the two edges (u, w) and (w, v) with a single edge (u, v).

In a network on X, each tree node has exactly one incoming edge, while each reticulation node has two or more incoming edges. If we remove all but one of the incoming edges for each reticulation node in N, the resulting network is a tree D with the same root in which the original reticulation nodes become nodes of in-degree 1 and out-degree 1 and some tree nodes become new leaves. If we delete all nodes that are not an ancestor of any leaf in X and suppress all nodes of in-degree and out-degree 1 in D, we obtain a tree T on X. We say that T is a tree *hard-displayed*, or simply *displayed*, in N.

In general, we say that a tree T' on a subset of taxa $X' \subseteq X$ is *displayed* in a network N on X if there exists a tree T on X that is displayed in N and T' can be obtained from T by removing all nodes (and associated edges) that are not ancestors of any leaf in X' and then suppressing all nodes of in-degree 1 and out-degree 1.

B.2 Soft-display of trees

Let T be a tree on X. A *split* of a multifurcation u in T involves inserting a new node w into an outgoing edge (u, v), and reattaching at least one other child of u, different from v, to w. In reverse, let $e = (u, v)$ be an edge of T, *contracting* e involves deleting e and combining u and v into a single node w so that all edges incident to u or v (except e) now become incident to w.

A tree T' is said to be a *refinement* of T if there is a series of trees $T_1, T_2, \cdots, T_k$ such that (i) $T_1 = T$, (ii) $T_k = T'$, and (iii) T_i is obtained from T_{i-1} by applying a split operation for each $2 \leq i \leq k$.

A tree T on $X' \subseteq X$ is *soft-displayed* in a network N on X if a refinement of T is displayed in N.

B.3 Inference of networks

Note that a network on X can display multiple trees on X. An approach of inference of a network on X is formulated as the following algorithmic problem:

> **Input**: A family of k trees T_i on X_i.
>
> **Output**: A network on $X = \cup_{1 \leq i \leq k} X_i$ with the minimum hybridization number that hard-displays (resp. soft-displays) all the input trees.

The network space is vast, making it extremely challenging to design efficient algorithms for related problems. In this study, we focus on finding a network with the minimum hybridization number in the space of tree-child networks.

C Encoding trees into (hyper-)sequences

C.1 Lineage taxon sequences and hypersequences

Let X be a taxon set. A *lineage taxon sequence* (LTS) is a finite ordered sequence (i.e., string) of elements from X. The empty taxon sequence is the unique sequence over X that contains no taxa. For a taxon x and an LTS s, we use $|s|_x$ to denote the number of times x appears in s. The *length* $|s|$ of s is defined as $\sum_{x \in X} |s|_x$.

A *lineage taxon hypersequence* (LTH) is a finite ordered sequence of subsets of X. The empty taxon hypersequence is the unique sequence that contains no subsets. For a subset S of X and an LTH h, we use $|h|_S$ to denote the number of times S appears in h. The *length* $|h|$ of h is defined as $\sum_{S \subseteq X} |h|_S$. We also define the *norm* $\|h\|$ of h as $\sum_{x \in X} |h|_x$.

C.2 LTS encoding and decoding for binary trees

Let X be an ordered set of n taxa where

$$x_1 < x_2 < \cdots < x_n.$$

Under the ordering, each *binary* tree on X corresponds one-to-one to a set of n sequences, one for each taxon, called *the lineage taxon sequences* of the taxa.

Let T be a binary tree on X. For each internal node u in T, we define:

$$m(u) = \min\{x \in X : x \text{ is the label of a leaf below } u\}.$$

We map T to a set containing n sequences (which may be empty) using the following encoding algorithm.

Algorithm C.1 (LTS Encoding Algorithm)**.**

Input: A *binary* tree on X where the n taxa are ordered as $x_1 < x_2 < \cdots < x_n$.
Output: One LTS per taxon.

1. Label the root ρ with the smallest taxon $\lambda(\rho) = x_1$.
2. Label every internal node u, with children v and w, with $\lambda(u) = \max\{m(v), m(w)\}$, the larger of the smallest taxon labels below v and below w.
3. For each taxon x_i, we extract its LTS by listing the labels along the path from the unique internal node labeled x_i to the leaf x_i.

Note that in this paper, the LTS of x_i starts and ends with x_i, which differs from the definition presented in [Zhang et al., 2023], where x_i is not explicitly included in its LTS.

The above encoding algorithm is illustrated in Fig. 3b and c. The LTSs of the n taxa output by the LTS Encoding Algorithm for a binary tree T satisfy the properties listed in Lemma 1 in the main text. Conversely, one can recover the tree T from the corresponding LTSs of the taxa using the following decoding algorithm, illustrated in Fig. 3d.

Algorithm C.2 (LTS Decoding Algorithm for Binary Trees)**.**

Input: One LTS per taxon.
Output: The corresponding rooted, binary phylogenetic tree.

1. For each LTS of x_i, construct a path P_i by creating a node for each symbol in the LTS: Connect each node to the next one if it appears in the middle of the path. Label the final node with x_i.
2. For each i, connect the unique node corresponding to the taxon x_i (which lies in the middle of some path P_j with $j < i$) to the first node of the path P_i.
3. Suppress the nodes that were the starting nodes of the paths P_i.

Note that we consider a node to be "in the middle of a path" if it contained in the path and is neither the first nor last node in the path.

C.3 LTH encoding and decoding for unrestricted trees

Assume that the elements of X are ordered as:

$$x_1 < x_2 < \cdots < x_n.$$

Given an arbitrary tree T on X, we will derive an LTH for each taxon by labeling internal nodes. Recall that $m(u)$ denotes the minimum label below an internal node u in T

The root is always labeled with the smallest taxon $\{\pi_1\}$.

For each internal node u with two children v_1 and v_2 in T, there is the minimum taxon $m(v_1)$ in the subtree below v and the minimum taxon $m(v_2)$ in the subtree below v_2. We will label v with the larger of $m(v_1)$ and $m(v_2)$, which is $\max(m(v_1), m(v_2))$.

In general, for a non-binary internal node u with k children $v_1, v_2, \cdots, v_k$, u is labeled with the subset consisting of all but the smallest taxon in $\{m(v_1), m(v_2), \cdots, m(v_k)\}$, which is
$\{m(v_1), m(v_2), \cdots, m(v_k)\} - \min_{1\leq i\leq k} m(v_i)$ (see Fig. 3a).

Remarks. (i) When an ordering on X is given, the node labeling of a tree on X is unique.

(ii) We may consider the label of a binary node as a subset containing a single element. In this way, each element appears in exactly one labeling subset.

Given an order of taxa, we label all internal nodes using the procedure described above. We use $\lambda(u)$ to denote the label of u for an internal node u. For each taxon t, there is a unique internal node u_t whose label subset contains t. Let the path P from u_t to the leaf representing taxon t be:

$$u_t = v_1, v_2, \cdots, v_k = t.$$

We call $t\lambda(v_2)\cdots\lambda(v_{k-1})t$ the LTH of t.

The LTH Encoding Algorithm is illustrated in Fig. 3a and b and summarized as follows.

Algorithm C.3 (LTH Encoding Algorithm)**.**

Input: A (not necessarily binary) tree on X where the n taxa are ordered as $x_1 < x_2 < \cdots < x_n$.
Output: One LTH per taxon.

1. Label the root ρ with the smallest taxon $\lambda(\rho) = x_1$.
2. Label every internal node u with k children v_i with the subset

$$\lambda(u) = \{m(v_i) \mid 1 \leq i \leq k\} - \min_{1\leq i\leq k} m(v_i).$$

3. For each taxon x_i, we extract its LTH by listing the label subsets along the path from the unique internal node whose label subset S contains x_i to the leaf x_i and then replacing S with x_i.

Proposition C.4. *Let T be an arbitrary tree on an ordered set X where its n taxa are ordered as $x_1 < x_2 < \cdots < x_n$. Let LTH(x_i) denote the LTH of x_i. Then, the LTHs obtained using Algorithm C.3 have the following properties:*

(i) For each i, the sequence LTH(x_i) has x_i as both its first and last element.

(ii) For each $i > 1$, there exists a unique $j < i$ such that x_i is an element in some labeling subset appearing in LTH(x_j).

Proof. For the binary tree case, the proof can be found in the supplementary material of [Zhang et al., 2023]. The argument can be easily generalized to the case of unrestricted trees. □

Since each LTH uniquely defines a path in the tree and the labels of internal nodes allow one to "glue" the paths together to recover the tree, the set of LTHs under an ordering uniquely define the tree. Such a decoding algorithm is illustrated in Fig. 3d and is summarized below.

Algorithm C.5 (LTH Decoding Algorithm I)**.**

Input: A set of LTHs satisfying the properties in Proposition C.4.
Output: The corresponding rooted phylogenetic tree.

1. For each LTH(x_i), construct a path P_i by creating a node for each symbol in the LTH:
   - Connect each node to the next one if it appears in the middle of the path.
   - Label the final node with x_i.
2. For each i:
   - connect the unique node corresponding to a subset containing x_i (which lies in the middle of some path P_j with $j < i$) to the first node of the path P_i.
3. Suppress the nodes that were the starting nodes of the paths P_i.

C.4 LTH decoding for tree-child networks

In this study, we focus on tree-child networks. One reason is that we can construct a tree-child network from the LTHs, with certain constraints to be specified later. Algorithm C.5 can be generalized to construct a tree-child network in the following way.

Algorithm C.6 (LTH Decoding Algorithm II)**.**

Input: A set of LTHs satisfying the properties in Proposition C.7.
Output: The corresponding tree-child network.

1. For each LTH(x_i), construct a path P_i by creating a node for each symbol in the LTH:
   - Connect each node to the next one if it appears in the middle of the path. Label the final node with x_i.
2. For each i:
   - Connect *every* node corresponding to a subset containing x_i (which lies in the middle of some path P_j with $j < i$) to the first node of the path P_i.
3. Suppress the nodes that were the starting nodes of the paths P_i *if it has in-degree 1 and out-degree 1.*

This algorithm is illustrated in Fig. 5d.

Proposition C.7. *Let X be a taxon set with n taxa ordered as $x_1 < x_2 < \cdots < x_n$. Assume that there are n LTHs, denoted LTH(x_1), LTH(x_2), $\cdots$, LTH(x_n), each corresponding to one of the n taxa in X. Suppose the LTHs satisfy the following conditions for each $1 \leq i \leq n$:*

(i) LTH(i) starts and ends with x_i.

(ii) Only elements in the subset $\{x_i, \cdots, x_n\}$ can appear in LTH(x_i).

(iii) The element x_i appears in at least one LTH(x_j) with $j > i$.

Then, given these LTHs as input, Algorithm C.6 constructs a tree-child network on X with hybridization number $\sum_{1 \leq i \leq n} \|LTH(x_i)\| - 3|X| + 1$.

Proof. If we draw the n paths $P_1, P_2, \cdots, P_n$ horizontally and arrange them from top to bottom (see Fig. 5d), then the digraph N produced by the algorithm is acyclic. This is because each arc within a path P_i is oriented from left to right, and each inter-path arc is oriented downward–from a node in P_j to a node in P_i with $j < i$.

According to the property (iii), there is always an arc from a node in P_j to the first node of P_i with $j < i$ for each i. Thus, N is connected.

The tree-child property of N follows from the fact that, for $j > 1$, only the first node of the path P_j can be a reticulation node, while all subsequent nodes are tree nodes in N.

For each $i > 1$, the number m_i of arcs entering the first node f_i of P_i is equal to the total numbers of times x_i appears in all $P_1, P_2, \cdots, P_{i-1}$, Thus, if the m_i is equal to 1, f_i will be contracted and thus is invisible in N. If m_i is larger than 1, f_i is a reticulation node of N with in-degree m_i. Thus, the hybridization number of N is $\sum_{1 \leq i \leq n} m_i = \|\text{LTH}(x_i)\| - 3|X| + 1$, as each taxon is also the first and the last taxon of LTH(x_i) and x_1 has in-degree 0. □

D PhyloFusion approach

PhyloFusion is an extension of the ALTS algorithm. It takes a set of unrestricted trees on different taxa and computes a tree-child network that displays the input trees while heuristically aiming to minimize the hybridization number.

D.1 Shortest common super-hypersequence (SCSH)

A taxon sequence s is considered a *supersequence* of another taxon sequence s' if s' is identical to s or s' can be obtained from s by removing one or more symbols.

Similarly, a taxon hypersequence $x = x_1 x_2 \cdots x_n$ is considered a *super-hypersequence* of another taxon sequence $y = y_1 y_2 \cdots y_m$ if there are m indices with $i_1 < i_2 < \cdots < i_m$ and x_{i_k} is a superset of y_k for each $1 \leq k \leq m$. For instance, $b\ \{c, d, e\}\ e\ c\ b$ is a super-hypersequence of $b\ \{c, e\}\ b$.

The *SCSH problem* is, given a set of taxon hypersequences, to find a shortest taxon hypersequence that is a super-hypersequence of every given hypersequence. This algorithmic problem is NP-hard. This is because the shortest common supersequence (SCS) problem is a restricted case of the SCSH problem.

D.2 Exact algorithm

Algorithm D.8 (Exact Inference Algorithm)**.**

Input: Trees $T_1, \ldots, T_k$ on (not necessarily identical) taxon sets $X_1, \ldots, X_k$, respectively.
Output: Tree-child network N on $\cup_{i=1}^k X_i$ with minimum hybridization number,
that hard-displays every input tree T_i.
1. Set $X = \cup_{i=1}^k X_i$.
2. For each order $\mathcal{O}$ on X: $x_1 < x_2 < \cdots < x_n$:
   2.1 For each $j = 1, \ldots, k$:
      For each $i = 1, \ldots, n$:
         if x_i appears in T_j, compute $\mathrm{LTH}_j(x_i)$ using Algorithm C.3.
         Otherwise, set $\mathrm{LTH}_j(x_i) = x_i x_i$.
   2.2. For each $i = 1, \ldots, n$:
      Compute a SCSH S_i for $\mathrm{LTH}_1(x_i), \mathrm{LTH}_2(x_i), \cdots, \mathrm{LTH}_j(x_k)$.
   2.3. Construct a tree-child network $N_{\mathcal{O}}$ from $\{s_i \mid 1 \le i \le k\}$ using Algorithm C.6.
3. Output the network with the minimum hybridization number constructed in Step 2.

Theorem D.9. *Given k trees $T_1, \ldots, T_k$ on $X_1, \ldots, X_k$ as input, Algorithm D.8 produces a tree-child network on $X = \cup_{i=1}^k X_i$ with the minimum hybridization number that hard displays all the input trees.*

Proof. Clearly, the algorithm constructs a tree-child network. Now, consider an order $\mathcal{O}$ on X:

$$x_1 < x_2 < \cdots < x_n.$$

We now fix a tree T_j and show that it is hard displayed in $N_{\mathcal{O}}$ produced in Step 2.3.

For each taxon x_i, by assumption, S_i is a super-hypersequence of $\mathrm{LTH}_j(x_i)$ of x_i in T_j. Without loss of generality, we may assume $S_i = s_{i1} s_{i2} \cdots s_{i\ell_i}$, where ℓ_i is the length of S_i, and

$$\mathrm{LTH}_j(x_i) = x_{i1} x_{i2} \cdots x_{it}$$

such that

$$\begin{gathered} s_{i1} = x_{i1} = x_i, \\ x_{im} \subseteq s_{ik_m} \subset X, \ m = 2, 3, \cdots, t-1, \\ s_{i\ell_i} = x_{it} = x_i. \end{gathered}$$

for a sequence of t indices $k_1 < k_2 < \cdots < k_t$.

Also note that each node of the constructed path P_i corresponds one-to-one to a subset of S_i. For each s_{im}, the node p_{im} has $|s_{im}| + 1$ outgoing edges. We remove some edges from $N_{\mathcal{O}}$ by considering the following cases:

- If $s_{im} \notin \{s_{ik_1}, s_{ik_2}, \cdots, s_{ik_t}\}$, we remove all the inter-path arcs leaving p_{im}, the node corresponding to s_{im}.

- If $s_{im} \in \{s_{ik_1}, s_{ik_2}, \cdots, s_{ik_t}\}$, say $s_{im} = s_{ik_q}$ for some $q \le t$, then we remove the inter-path arcs defined by the elements in $s_{ik_q} \setminus x_{iq}$.

The resulting subtree is a subdivision of the tree T_j and thus (hard-)displays it.

Lastly, let N be a tree-child network with the minimum hybridization number that (hard-)displays all input trees T_j. We prove that there is an order $\mathcal{O}$ on X with which the network produced in Step 2.3 has the same hybridization number as N and (hard-)displays all the trees.

Let N have k reticulation nodes. Assume that we list the root ρ of N and the k reticulation nodes as

$$r_0 = \rho, r_1, \cdots, r_n$$

such that for any r_i and r_j, $i < j$ if a parent of r_j lies in the tree component C_i rooted at r_i. (Tree components of a tree-child network are defined in Section A.2.) This ordering of reticulation nodes can be obtained by performing a topological sort of all the nodes in N.

We then order the leaves of N by listing the leaves in the tree component rooted at r_0, followed by those rooted at r_1, and so on. Let the resulting leaf order be denoted by $\mathcal{O}$.

With the order $\mathcal{O}$, we can label the tree nodes of N as follows.

1. Label the reticulation node r_i with the smallest taxon in the tree component rooted at r_i.
2. For each internal tree node u with children $v_1, v_2, \cdots, v_t$ in a tree component C_j, we define $m(v_i)$ as the smallest leaf below v_i in C_j if v_i is in C_j and to be the label of v_i if v_i is a reticulation node. We then label u with the subset $\{m(v_1), m(v_2), \cdots, m(v_k)\} - \min_{1 \le i \le k} m(v_i)$, which is the set of $m(v_i)$ values excluding the minimum.

Similar to the case of labeling trees, we extract an LTH for each taxon x_i by listing the label subsets along the path from the unique tree node whose label subset S contains x_i to the leaf x_i and then replacing S with x_i. We use $\text{LTH}_N(x_i)$ to denote this LTH.

Since each tree T_j is displayed in N, $\text{LTH}_N(x_i)$ is a super-hypersequence of the LTH in the tree for each taxon x_i. Since the hybridization number of N is equal to $\sum_{1 \le i \le n} \|\text{LTH}_N(x_i)\| - 3|X| + 1$, $\text{LTH}_N(x_i)$ has to be the shortest common super-hypersequence of the LTHs of x_i in all the input trees. Otherwise, we could use the related shortest common super-hypersequences to produce a tree-child network with a hybridization number smaller than N. Note that when a shortest common super-hypersequence S of the LTHs for each taxon x_i is computed in Algorithm D.11, then the objective function to be minimized is $\|S\|$.

This concludes the proof of the theorem. □

D.3 Cluster-based reduction rule for simplification

In this subsection, we recap a cluster-based reduction rule (see Fig. 7) used to accelerate the network inference process, which has been adopted by various programs [Albrecht, 2015, Huson and Linz, 2018, Zhang et al., 2023].

Let u be an internal node in a tree T. The node cluster $C(u)$ of u is defined as the set of taxa descended from u. The subtree $T(u)$ induced by u consists of u, all its descendants, and the arcs connecting them.

A set of trees on X is said to be reducible if they share a common node cluster; otherwise, it is irreducible. Clearly, if multiple trees share a common node-induced subtree, they are reducible.

Let S consist of k trees $T_1, T_2, \cdots, T_k$, on taxon sets $X_1, X_2, \cdots, X_k$, respectively. Assume that T' is a maximal common node-induced subtree of these trees. Let y be a new (formal) taxon that is not contained in $\cup_{1\leq i\leq k} X_i$. For $i = 1, \ldots k$, let $u_i \in X_i$ denote the node for which $T_i(u_i)$ equals T'. By replacing $T_i(u_i)$ by y, we obtain a new tree T'_i on $\{y\} \cup X_i \setminus C(u_i)$. Let S' denote the set of trees obtained from S in this way. Clearly, replacing the taxon with label y with the T' in a tree-child network that displays the trees in S', we obtain a tree-child network with the same hybridization number that displays the trees in S. The process of obtaining S' from S is called a *subtree-reduction* [Bordewich and Semple, 2005].

More generally, assume that the trees in S (as defined above) have a minimal node cluster A of size ≥ 2 in common. Let y be a new (formal) taxon that is not contained in any of the taxon sets. For $i = 1, \ldots k$, let $u_i \in X_i$ denote the node for which $C(u_i) = A$. By replacing $T_i(u_i)$ by y, we obtain a new tree T'_i on $\{y\} \cup X_i \setminus A$. Let S' denote the set of all trees obtained in this way. Moreover, set $S'' = \{T_1(u_1), T_2(u_2), \cdots, T_k(u_k)\}$.

In this way, we decompose S into two sets of trees: S' and S''. Assume that we have a tree-child network N' on $(\{y\} \cup X_1 \cup X_2 \cdots \cup X_k) \setminus A$ that displays all trees in S' and a network N'' on A that displays all trees in S''. Then, the tree-child network obtained from N', by replacing y with N'', displays all trees in S [Baroni et al., 2006]. This process is called the cluster-reduction rule.

D.4 Refinement rule for multifurcations

Let the taxa of X be ordered as:

$$x_1 < x_2 < \cdots < x_n.$$

We consider k trees:

$$T_1, T_2, \cdots, T_k.$$

Let the LTH S_{ij} of x_i in T_j be:

$$S_{ij} = x_i s_1^{(ij)} s_2^{(ij)} \cdots s_{\ell_{ij}}^{(ij)} x_i,$$

where $1 \leq i \leq n, 1 \leq j \leq k$.

We consider each taxon from x_1 to x_{n-2} sequentially. For j from 1 to k, we apply the following refinement rule.

> (**Refinement Heuristic**) For t from 1 to ℓ_{ij}, if $s_t^{(ij)}$ is a subset containing a taxon x_k (where $k \geq i+1$) and a subset R such that (i) all the taxa in R do not appear in $S_{it''}$ for any $t'' \neq t$ but (ii) they appear in $S_{kt'}$ for some t'. Then, we update $s_t^{(ij)}$ and S_{tj}:
>
> $$s_t^{(ij)} \leftarrow s_t^{(ij)} \setminus R, \;\; S_{kj} \leftarrow x_k R \diamond S'_{kj},$$
>
> where S'_{kj} is an LTH obtained from S_{kj} by removing x_k from the front and $\diamond$ is the concatenation operation for strings.

The above rule is illustrated in Figure 8. Applying the rule is equivalent to splitting the non-binary node w, originally labeled with $s_t^{(ij)}$, into two nodes in T_j: w is relabeled with the updated $s_t^{(ij)}$, and a new node labeled with the subset R is inserted along the path from w to the leaf labeled x_k.

D.5 Add-leaf rule for missing taxa

We have the same assumption on the ordering of the taxon set and the LTHs of taxa in each input tree as in Section D.4. We use the following rule to deal with the missing taxa:

> (**Add-leaf heuristic**) For each pair of indices j and k such that (1) the label subset $s_t^{(ij)}$ in S_{ij} contains a taxon a that is missing in the tree T_k and (2) S_{ik} contains a taxon b that appears in the LTH of a in the tree T_j. Then, we update S_{ik} by replacing b with a and set the LTH of a in tree T_k to aba (Fig. 9).

The conditions (1) and (2) imply that $x_i < a < b$ holds, with respect to the ordering of X. Therefore, the updating operation is equivalent to inserting the taxon a in the edge from the internal node labeled with x_i to the node labeled with the first symbol $s_1^{(ik)}$ of S_{ik} (if S_{ik} is not empty) and the leaf b otherwise.

References

Benjamin Albrecht. Computing all hybridization networks for multiple binary phylogenetic input trees. *BMC Bioinformatics*, 16:236, 2015. doi: 10.1186/s12859-015-0660-7.

Mihaela Baroni, Charles Semple, and Mike Steel. Hybrids in real time. *Systematic Biology*, 55(1):46–56, 2006. doi: 10.1080/10635150500431197.

M. Bordewich and C. Semple. On the computational complexity of the rooted subtree prune and regraft distance. *Annals of Combinatorics*, 8(4):409–423, 2005.

Gabriel Cardona, Mercè Llabrés, Francesc Rosselló, and Gabriel Valiente. Comparison of tree-child phylogenetic networks. *IEEE/ACM Transactions on Computational Biology and Bioinformatics*, 6(4):552–69, 2009. doi: 10.1109/TCBB.2007.70270.

Péter L. Erdős, Charles Semple, and Mike Steel. A class of phylogenetic networks reconstructable from ancestral profiles. *Mathematical Biosciences*, 313:33–40, 2019. doi: 10.1016/j.mbs.2019.02.003.

D.H. Huson and S. Linz. Autumn algorithm—computation of hybridization networks for realistic phylogenetic trees. *IEEE/ACM Transactions on Computational Biology and Bioinformatics*, 15:398–420, 2018.

D.H. Huson, R. Rupp, and C. Scornavacca. *Phylogenetic Networks*. Cambridge, 2012.

Louxin Zhang, Niloufar Abhari, Caroline Colijn, and Yufeng Wu. A fast and scalable method for inferring phylogenetic networks from trees by aligning lineage taxon strings. *Genome Research*, 33(7):1053–1060, 2023.
